# Talking about firearm injury prevention with patients: a survey of medical residents

**DOI:** 10.1186/s12909-021-03024-9

**Published:** 2022-01-03

**Authors:** Rocco Pallin, Sara Teasdale, Alicia Agnoli, Sarabeth Spitzer, Rameesha Asif-Sattar, Garen J. Wintemute, Amy Barnhorst

**Affiliations:** 1 Violence Prevention Research Program, Department of Emergency Medicine, University of California Davis School of Medicine, 2315 Stockton Blvd, Sacramento, CA 95819 USA; 2 University of California Firearm Violence Research Center at UC Davis, 2315 Stockton Blvd, Sacramento, CA 95819 USA; 3 Department of Emergency Medicine, University of California, Davis, 2315 Stockton Blvd, Sacramento, CA 95817 USA; 4 Department of Internal Medicine, University of California, Davis, 2315 Stockton Blvd, Sacramento, CA 95819 USA; 5 Department of Family and Community Medicine, University of California, Davis, 2315 Stockton Blvd, Sacramento, CA 95819 USA; 6 Department of General Surgery, Brigham and Women’s Hospital, 75 Francis Street, Carrie Hall 103, Boston, MA 02115 USA; 7 Department of Psychiatry and Behavioral Sciences, University of California, Davis, 2315 Stockton Blvd, Sacramento, CA 95819 USA

**Keywords:** Medical education, Firearm injury prevention, Patient counseling, Medical interns, Medical training, Firearm violence, Safe fiream storage

## Abstract

**Background:**

Firearm injury and death are significant public health problems in the U.S. and physicians are uniquely situated to help prevent them. However, there is little formal training in medical education on identifying risk for firearm injury and discussing safe firearm practices with patients. This study assesses prior education, barriers to counseling, and needs for improved training on firearm safety counseling in medical education to inform the development of future education on clinical strategies for firearm injury prevention.

**Method:**

A 2018 survey administered to 218 residents and fellows at a large, academic medical center asked about medical training on firearm injury prevention, frequency of asking patients about firearm access, and perceived barriers.

**Results:**

The most common barriers cited were not knowing what to do with patients’ answers about access to firearms (72.1%), not having enough time (66.2%), not feeling comfortable identifying patients at risk for firearm injury (49.2%), and not knowing how to ask patients about firearm access (48.6%). Prior education on firearm injury prevention was more strongly associated with asking than was personal exposure to firearms: 51.5% of respondents who had prior medical education reported asking compared with who had not received such education (31.8%, *p*=0.004). More than 90% of respondents were interested in further education about interventions, what questions to ask, and legal mechanisms to separate dangerous people from their firearms.

**Conclusions:**

Education on assessing risk for firearm-related harm and, when indicated, counseling on safe firearm practices may increase the likelihood clinicians practice this behavior, though additional barriers exist.

**Supplementary Information:**

The online version contains supplementary material available at 10.1186/s12909-021-03024-9.

## Background

Firearm injury and death are significant public health problems in the United States, and physicians occupy a unique position to address it. In 2019, 39,704 people died by firearms, nearly two-thirds of them from firearm suicides [1]. Firearms are readily accessible to many; an estimated 35% of American adults live in a household with a firearm, and in a majority of those households firearms are not stored in the most secure manner [2, 3]. A substantial body of evidence has found that firearms in the home increase the risk of firearm suicide, homicide, and unintentional injury for those living there, and that safe firearm storage reduces the risk of firearm-related harm [4–10]. By knowing how to identify risk for firearm-related harm, knowing possible steps to reduce access by at-risk persons, and talking with patients, parents, or caregivers about the danger of access to firearms in the home when someone is at increased risk, clinicians can help prevent firearm injury and death [11].

Despite this, many clinicians do not routinely ask about access to firearms in their practice, even when such screening is clinically indicated [12, 13]. One study of emergency department patients with suicidal ideation or attempt found that 55% of the patients discharged home had no documentation of a lethal means assessment, and 13% of those patients later told researchers they had at least 1 firearm at home at the time [14]. In a survey of practicing psychiatrists, only 27% of respondents said they routinely asked their patients about firearm safety, although most believed that their patients were at higher risk of adverse events involving firearms [15]. A survey of family practice physicians and pediatricians found that, despite believing they had a responsibility to do so, very few counselled patients about safe firearm practices [16].

Physicians cite lack of knowledge as a primary barrier to firearm counseling in a clinical setting [12]. Groups such as the American College of Physicians [17], California Medical Association [18], and American Academy of Pediatrics [19] support physician screening for firearm violence prevention, and research suggests that 65 to 93 percent of physicians recognize firearm counseling as within a physician’s scope of practice [20]. Physicians may need education and training on firearm injury prevention in order to incorporate it into their practices. To our knowledge, few medical schools and residency training programs include substantial formal training on discussing firearms with patients in their core curriculum.

A 2016 review of the literature on firearm safety curricula in medical education found only two reports evaluating programs teaching medical students or residents [21]. Two articles in Academic Psychiatry in 2010 called for better training on anticipatory guidance related to firearms in psychiatric residency [22, 23], but five years later, a literature review found no such training directed at psychiatrists or psychiatry residents [21]. In the 1990s, only one third of pediatric residency programs nationwide offered any firearm safety counseling training, and only 16 percent of family practice residencies offer such training [24, 25]. Given that practitioners who had received information about firearm safety were more likely to have such discussions with their patients [15], it has been suggested that the development of firearm safety education programs for physicians be made a priority [21].

To examine specific needs for improved training, we conducted a survey to assess to what degree and by what methods medical trainees at a major academic medical center are taught about firearm injury prevention, and to what extent they are implementing that knowledge in their clinical practice. The survey also explored whether trainees are having these discussions with patients, why they are not, and what they would like to be taught on this topic.

## Methods

### Study population

During a two-week period in March and April of 2018, we distributed an electronic survey via email to all 759 residents and fellows at a large, urban, university-based academic medical center.

### Survey instrument

We developed a 12-question anonymous survey. We performed a literature review, created a question bank, and, where possible, adopted questions used in prior surveys on firearm injury prevention in medical education. The survey questions addressed what trainees had been taught about firearms thus far in their medical education, if they asked about firearm access in clinical practice, what had been their personal experience with firearms, and their interest in firearm injury education, as well as basic demographic information. Using a 5-point scale, residents answered questions assessing frequency of screening for firearm injury prevention, exposure to firearms over their lifetimes, and interest in education programs designed to increase knowledge. The survey instrument was not validated. The full survey instrument is available in Additional file 1.

### Data collection

The graduate medical education office electronically distributed the link to the survey, along with a cover letter explaining the purpose of the study, to all residents and fellows twice over a 2-week period.

We administered the survey online using Qualtrics. All participants were provided an informed consent statement, including that their participation was voluntary and responses anonymous, before beginning the survey. Survey initiation constituted consent.

### Data analysis

We conducted bivariate analyses using the χ^2^ test for significance and used logistic regression to calculate odds ratios with 95% confidence intervals. When cell sizes were small (n<5), Fisher’s exact tests were used to obtain p values. All analyses were conducted using Stata SE 15.1 for Mac (StataCorp, College Station, Texas).

We combined respondent specialties for analysis to yield five aggregate specialty groups: primary care, surgery, emergency, psychiatry, and other. The primary care group included respondents from family medicine, pediatrics, internal medicine, and the psychiatry/family medicine joint program to reflect the Association of American Medical Colleges designation, as well as obstetrics and gynecology. We collapsed responses to Likert or 5-point scale questions for analysis.

We classified all respondents as having either no or some lifetime firearm exposure based on their responses to questions on topics such as involvement in firearm safety courses, current or past firearm ownership, and exposure to firearms in the military.

The University of California at Davis Institutional Review Board deemed that this study did not constitute research and waived the need for ethics approval. All data collection and analyses were carried out in accordance with relevant reporting guidelines and regulations.

## Results

Of the 759 residents and fellows who received the survey link, 218 (28.7%) completed the survey. Most respondents (83.9%) were residents, 54.6% were female, 85.8% were between the ages of 25 and 34, and 50.7% were in primary care (Table 1).

**Table 1:** Description of survey respondents (*n* =218)

	Count	Percent
TOTAL	225	-
Level of training		
Resident	183	83.9
Fellow	35	16.1
Gender		
Male	98	45.0
Female	119	54.6
Other/Prefer not to say	1	0.5
Age		
25-34	188	85.8
35-44	26	11.9
45-54	5	2.3
Specialty category		
Primary Care^*a*^	104	50.7
Surgery	33	16.1
Emergency	24	11.7
Psychiatry	20	9.8
Other	24	16.1
Exposure to guns		
Some lifetime exposure to firearms^*b*^	104	47.5
Took a safety course	49	22.4
Owns a firearm	30	13.7
Used a firearm in the military	9	4.1
Lives in a gun household	16	7.3
Grew up in a house with guns	67	30.6
Fired a gun in the last year	44	20.1
Some lifetime exposure to guns by gender		
Male	56	57.1
Female	47	39.5
Had education on firearms in medical education	132	60.0
State attended high school		
California	116	53.5
Other state	90	41.5
International	11	5.1

^a^Primary care specialty includes respondents from family medicine, pediatrics, obstetrics and gynecology, and internal medicine. If respondents were in psychiatry/primary care joint program, they were included in the primary care group for these analyses

^b^Excluding training in medical education. See Additional file 2 for definition of lifetime exposure to firearms

Nearly half of respondents (47.5%) reported at least one lifetime exposure to firearms, including 30.6% who grew up in homes with firearms. More than half (60.0%) reported having had education on firearms during their medical training (Table 1). See Additional file 2 for definition of collapsed variable categories.

Respondents with lifetime firearm exposure more often reported asking (49.0%), compared with those having no lifetime firearm exposure (39.1%, *p*=0.140). Those whose medical education included the topic of firearms more often reported asking (51.5%) compared with those whose education had not covered firearms (31.8%, *p*=0.004). However, the groups were equally interested in future education on firearms (70.4 and 73.9%, respectively) (Table 2).

**Table 2:** Frequency of and barriers to asking about firearms by firearm exposure and by prior firearm education (percent)

	Lifetime exposure to firearms (% of respondents)		Firearm education in medical training (% of respondents)	
	Yes	No	Yes	No
Frequency of asking patients about firearms				
Never	51.0	60.9	48.5	68.2^a^
At least sometimes	49.0	39.1	51.5^a^	31.8^a^
Reasons for not asking about firearms^b^				
Not enough time	59.5	70.9	64.3	68.4
Afraid to offend patient	22.4^a^	40.2^a^	24.7^a^	43.3^a^
Don’t know if asking is legal	6.4^a^	24.6^a^	6.9^a^	27.5^a^
Nothing I can do if there is a gun in the home	30.0	24.7	28.2	27.0
Topic isn’t clinically relevant	28.3^a^	6.9^a^	14.8	21.6
Firearms aren’t within scope of practice	46.7	43.1	36.6^a^	54.8^a^
I don’t know how to ID risk	42.9	54.3	45.7	52.6
I don’t know how to ask	45.9	50.7	35.4^a^	65.5^a^
I don’t know what to do with answers	75.7	70.2	64.6^a^	82.2^a^
Interested in education^c^	70.2	73.9	70.4	73.9

^a^*p* < 0.05, Fisher's exact used when cell size < 5

^b^Percent shown is percent of respondents who reported each reason as a notable barrier to asking patients about firearms

^c^Percent shown is percent of respondents who reported being “moderately,” “very,” or “extremely interested”

Reported frequency of asking patients about firearms varied by specialty. Majorities of psychiatry (90.0%) and emergency medicine (54.2%) respondents reported ever asking (Table 2). Fewer than half of primary care (45.2%), surgery (24.2%), and other (4.2%) respondents reported ever asking (*p*=0.000). Relative to respondents in primary care, those in psychiatry were more likely to ask patients about firearms (OR: 10.9, CI: 2.4-49.5); those in surgery (OR: 0.4, CI: 0.2-0.9) and other specialties (OR: 0.1, CI: 0.0-0.4) were less so (Table 3). Differences by specialty in having had firearm injury prevention covered in medical education and in interest in firearm injury prevention education were generally not statistically significant.

**Table 3:** Respondent experience and interest in clinical strategies for firearm injury prevention

	Percent	Odds ratio	95% CI	P value
Ever ask about firearm access^a^				
Primary Care	45.2	ref	-	-
Surgery	24.2	0.39	0.16-0.94	0.036
Emergency	54.2	1.43	0.59-3.49	0.428
Psychiatry	90.0	10.92	2.41-49.46	0.002
Other	4.2	0.05	0.01-0.41	0.005
Had firearm injury prevention in medical education				
Primary care	62.5	ref	-	-
Surgery	51.5	0.26	0.29-1.40	0.264
Emergency	79.2	1.24	0.79-6.59	0.128
Psychiatry	75.0	1.00	0.61-5.34	0.289
Other	33.3	0.14	0.12-0.77	0.012
Interested in firearm injury prevention education^b^				
Primary care	77.9	ref	-	-
Surgery	54.6	0.34	0.15-0.78	0.011
Emergency	79.2	1.08	0.36-3.20	0.89
Psychiatry	85.0	1.61	0.43-5.97	0.48
Other	58.3	0.40	0.16-1.01	0.053

^a^Odds ratios compared ever asking with never asking

^b^Odds ratios compare being “moderately,” “very,” or “extremely” interested in firearm injury prevention education with being “not” or “slightly” interested

We asked respondents about their primary firearm access-related concern for their patients: suicide, homicide, or accidents. Respondents in primary care (46.6%), emergency medicine (62.5%), and psychiatry (85.0%) reported being most concerned about suicide, while a majority of those in surgery (54.6%) reported homicide as their greatest concern. Compared to those in primary care, pyschiatrists were significantly more likely to report suicide as their major concern (OR: 6.5, CI: 1.9-23.5) and surgeons were significantly more likely to report homicide as their major concern (OR: 9.1, CI: 3.7-22.7).

**Fig. 1:**
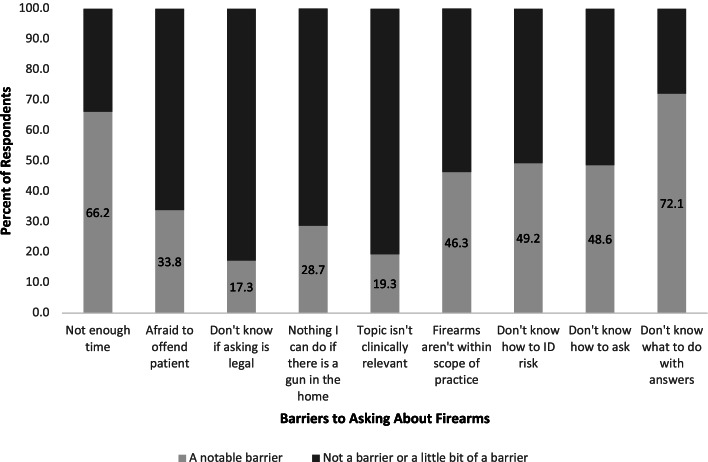
Percent of respondents reporting notable barriers to asking about firearms. Survey data from residents and fellows at a large, urban, university-based academic medical center in 2018 (*n*=218)

The most common barriers to asking patients about firearms that respondents cited were not knowing what to do with patients’ answers (72.1%), not having enough time (66.2%), not feeling comfortable identifying patients at risk for firearm injury (49.2%), and not knowing how to ask (48.6%) (Fig. 1). Psychiatry respondents reported notable barriers least often when compared with other specialties: 75% of psychiatry respondents reported insufficient time as the only notable barrier.

Respondents with lifetime exposure to firearms were less concerned about the legality of asking and less afraid of offending patients compared to those without exposure to firearms. Those with lifetime exposure to firearms more often did not ask because they did not think the topic was clinically relevant. Respondents with and without lifetime exposure were similar in reporting as notable barriers not knowing how to identify risk for firearm injury, not knowing how to ask, and not knowing what to do with the answers.

It might be worth noting that very few respondents in fact thought it might be illegal; this seems like it could be a generational difference between residents/ fellows and older trained physicians. Respondents who had received education less often believed that firearms are not within their scope of practice, and less frequently cited as barriers not knowing how to ask about firearms in the home and not knowing what to do with the answers patients gave about access to firearms.

Respondents were interested in education on addressing firearms with patients regardless of their prior training on firearm injury prevention. More than 90% were somewhat or very interested in an educational program designed to increase knowledge and skills in counseling patients in strategies for reducing risk of firearm injury. Specifically, 81.7% of respondents reported interest in evidence-based interventions to decrease firearm injury and death in their patients, 54.5% were interested in legal mechanisms to separate dangerous people from their firearms, and 48.8% were interested in what questions to ask at-risk patients about their access to firearms.

## Discussion

In light of increasing public health concern regarding firearm injury and death and a relative paucity of standard medical educational about firearms, this study examined the educational experience and behaviors related to risk of firearm access for patients and counseling when indicated among graduate medical trainees at a major academic medical center. Our findings suggest trainees endorse the clinician’s role in identifying patients at risk for firearm-related harm and, when clinically relevant, discussing strategies to reduce that risk and have broad interest in education on clinical strategies for preventing firearm-related harm. The results also shed light on a range of barriers, including time constraints and lack of knowledge of what to do when an at-risk patient has access to a firearm.

Respondents who had prior firearm education in medical training reported fewer barriers to addressing the topic in practice and a greater comfort in counseling asking than those who did not have firearm education in medical training. This suggests that medical education about firearms is effective in achieving its objective and might be a substantial driver of physician comfort and empowerment to raise this topic with patients. Variations between specialties were noted in several areas, including the frequency with which firearms were discussed and trainees’ primary concerns regarding firearms. In addition to general training at the level of medical school, specialty-specific medical education for trainees may be useful in addressing potential specialty-specific concerns. For example, psychiatrists are overwhelmingly more concerned about suicide compared with those in other specialties; this is appropriate, given the high-risk nature of their patient population. Training on firearm injury prevention for psychiatrists, and others who see patients at risk of suicide, could include information on assessing risk for suicide and understanding the importance of lethal means access for suicidal persons. Lethal means counseling—talking with patients to remove access to firearms, medications, and other lethal means of suicide—is a promising and increasingly prioritized component of suicide prevention [26–28]. Trainees who see patients at risk of suicide should be educated on the potential of temporary transfer of firearms, a common method of reducing lethal means, to lessen suicide risk [29].

Surgeons reported a primary concern for homicide; they see relatively few patients with life-threatening self-inflicted firearm injuries (most deaths from such injuries are at the shooting scene) [30]. Surgeons could be trained in reducing recurrent interpersonal injury, as risk for firearm injury is increased among those who have experienced firearm injury in the past [31, 32]. At some institutions, hospital-based violence intervention programs (HVIPs) support violently injured patients with comprehensive care that addresses both risk for future involvement in violence and psychological trauma, and provides social support to those who are recovering from violent injury [33].

Limited research and expert recommendations suggest that clinicians and trainees become educated on a harm reduction approach to firearm injury prevention, reasons for firearm ownership, the various safe firearm storage devices, firearms themselves, skills for having clinically relevant and collaborative conversations about the risks and benefits of firearms in the home, and strategies to keeping firearms out of the hands of those who are at risk [11, 13, 34–37]. Such conversations should include establishing a context of risk, focusing on reducing access to firearms for at-risk persons, and tailoring to the individual patient and storage or other risk reduction strategies that work for them.

A structured and standardized curriculum for trainees has the potential to decrease barriers to counseling, improve the practice of identifying risk for firearm-related harm, increase provider comfort with discussing the risks of access to firearms when appropriate, and knowing how to respond when an at-risk patients has firearm access. Currently, resources are available to assist in curriculum development and facilitate screening and counseling efforts. Several published articles [12, 13, 38] provide background information and specific recommendations. Other tools provide comprehensive online resources for learning about clinical strategies for preventing firearm-related harm, and make available tools for educators seeking to implement firearm injury prevention education in their curricula for medical and mental health clinicians and trainees [39, 40].

Our results should be interpreted with consideration of certain limitations. This study was a small, explorative survey done at a single academic medical center in California. Though we feel the responses generated are representative of the sentiments and behaviors of trainees at this institution, we are uncertain as to their generalizability to other institutions of medical training and other geographic settings. However, given the diversity of training backgrounds and medical specialties, we think this is an important contribution to the education science of firearm safety. The generalizability of our results may be further limited by the fact that our university has a dedicated firearm violence research center and, as a result, firearm injury prevention may be included in clinical education more often than at other institutions.

As with all survey research, our results are subject to non-response bias. Our survey had a response rate of 29%, and we believe this is due in part to our inability to incentivize survey participation. Non-respondents may be different than those who chose to participate and we do not have any data on non-respondents. As this was a small quality improvement survey for one institution, the survey instrument was not evaluated for validity or reliability prior to distribution.

## Conclusions

Clinicians occupy a unique position to identify increased risk for firearm-related harm and, when clinically indicated, to work with patients to reduce that risk. Many medical trainees, however, feel that their training inadequately addresses this topic and would like more education. Specialty, personal exposure to firearms, and previous education on the topic affected to what degree trainees discussed firearms with their patients, as well as their educational interests. Specialty-specific curricula that addresses specific concerns and barriers may be appropriate for residents and fellows.

## Supplementary Information

**Additional file 1.** The complete survey instrument.

**Additional file 2.** Definition of collapsed variable categories.

## Data Availability

The datasets used and/or analysed during the current study are available from the corresponding author on reasonable request.
