# Correction to: Talking about firearm injury prevention with patients: a survey of medical residents

**DOI:** 10.1186/s12909-022-03117-z

**Published:** 2022-02-24

**Authors:** Rocco Pallin, Sara Teasdale, Alicia Agnoli, Sarabeth Spitzer, Rameesha Asif-Sattar, Garen J. Wintemute, Amy Barnhorst

**Affiliations:** 1 Violence Prevention Research Program, Department of Emergency Medicine, University of California Davis School of Medicine, 2315 Stockton Blvd, Sacramento, CA 95819 USA; 2 University of California Firearm Violence Research Center at UC Davis, 2315 Stockton Blvd, Sacramento, CA 95819 USA; 3 Department of Emergency Medicine, University of California, Davis, 2315 Stockton Blvd, Sacramento, CA 95817 USA; 4 Department of Internal Medicine, University of California, Davis, 2315 Stockton Blvd, Sacramento, CA 95819 USA; 5 Department of Family and Community Medicine, University of California, Davis, 2315 Stockton Blvd, Sacramento, CA 95819 USA; 6 Department of General Surgery, Brigham and Women’s Hospital, 75 Francis Street, Carrie Hall 103, Boston, MA 02115 USA; 7 Department of Psychiatry and Behavioral Sciences, University of California, Davis, 2315 Stockton Blvd, Sacramento, CA 95819 USA

**Correction to: BMC Med Educ 22, 14 (2022)**

**https://doi.org/10.1186/s12909-021-03024-9**

Following publication of the original article [1], the authors would like to correct their article as follows:

1. The corresponding e-mail address has been changed from rspallin@ucdavis.edu to rspallin@berkeley.edu
2. Reference [40] has been added
3. Figure 1 has been updated. The correct figure is given below.
4. A few other minor corrections in the body of the text

**Fig. 1:**
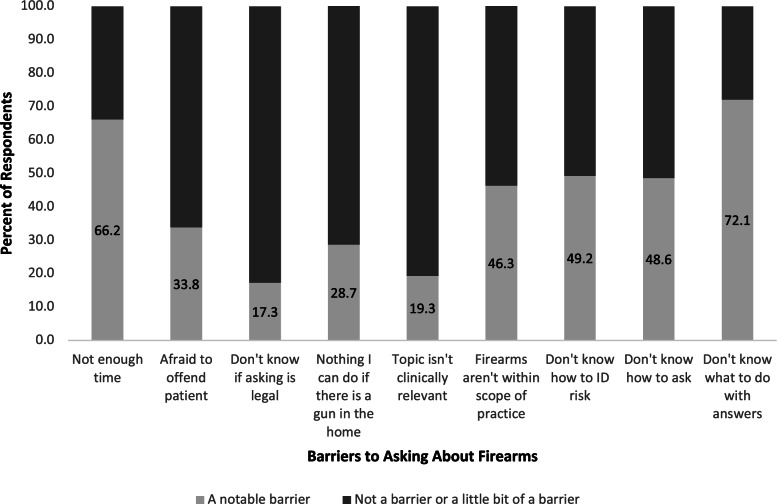
Percent of respondents reporting notable barriers to asking about firearms. Survey data from residents and fellows at a large, urban, university-based academic medical center in 2018 (*n* = 218)

The original article has been corrected.
