# Supplementary material for: Talking about firearm injury prevention with patients: a survey of medical residents
Source: BMC Med Educ. 2022 Jan 3;22:14. doi: 10.1186/s12909-021-03024-9 (PMC8725249; doi:10.1186/s12909-021-03024-9)
Supplement: Supplementary file 1 — Additional file 1. The complete survey instrument. [file 12909_2021_3024_MOESM1_ESM.pdf]

Additional File 1:

How often do you ask patients or their families about firearm injury prevention?

Never
Sometimes
About half the time
Most of the time
Always

When you do NOT ask patients about access to guns, to what degree do the following reasons keep you from asking?

	Not at all	A little	A moderate amount	A lot	A great deal
	0 10	20 30	40 50 60	70 80	90 100
I don’t have time.					
I’m worried that the patient will be offended.					
I’m not sure it’s legal for me to ask.					
There’s nothing I could do for a patient who is at risk for violence or suicide and has a gun.					
I don’t think gun access is a clinically relevant risk factor for injury or death.					
I don’t think asking is within my scope of practice.					
I don't feel comfortable identifying patients at risk for firearm injury.					
I don't know how to ask.					
I don't know what to do with the answers.					

In your patient population, are you more concerned about gun homicide, gun suicide, or accidental injury by a gun?

Homicide
Suicide
Accidental injury

When has firearm injury prevention been addressed in your medical education?

Never
Medical school
Residency or fellowship
Other professional education

How interested are you in an educational program designed to increase your knowledge and skills in counseling patients in firearm injury prevention?

Extremely interested
Very interested
Moderately interested
Slightly interested
Not interested at all

Which of the following would you be most be interested in learning more about? (pick 2)

How to identify patients at risk of firearm injury
What questions to ask at-risk patients about their access to guns
Evidence-based interventions to decrease firearm injury and death in my patients
When patients become prohibited from owning or purchasing guns
Legal mechanisms to separate dangerous people from their guns

Over the course of your life, what has been your involvement with firearms?

There were guns in the house where I grew up.
I used a firearm as part of my military service.
I have fired a gun in the last year.
I own at least one handgun, rifle or shotgun.
Someone else keeps guns in the house on or on the property where I live.
None of the above.

Have you ever taken a course in the safe handling and use of firearms (other than as part of military service)?

Yes
No
Not sure

Are you a medical student, resident, or fellow?

Medical student
Resident
Fellow

What did you complete you residency in?

What kind of fellowship are you doing?

What year of residency are you in?

1
2
3
4
5+

What is your specialty?

What year of medical school are you in?

1
2
3
4
5+

How old are you?

24 or younger
25 – 34
35 – 44
45 – 54
55 or older

In what state did you graduate high school? (This gives us an idea of where you grew up.)

What is your identified gender?

Male
Female
Other / Prefer not to answer
