# Supplementary material for: Talking about firearm injury prevention with patients: a survey of medical residents
Source: BMC Med Educ. 2022 Jan 3;22:14. doi: 10.1186/s12909-021-03024-9 (PMC8725249; doi:10.1186/s12909-021-03024-9)
Supplement: Supplementary file 2 — Additional file 2. Definition of collapsed variable categories. [file 12909_2021_3024_MOESM2_ESM.pdf]

Additional File 2:

Respondents were aggregated to the Primary Care specialty if they selected family medicine, obstetrics and gynecology, internal medicine, pediatrics, and psychiatry/family practice.

Respondents were aggregated to the Other specialty if they reported urology, anesthesiology, dermatology, diagnostic radiology/nuclear medicine, otolaryngology, pathology, physical medicine and rehabilitation, radiation oncology, diagnostic radiology, and neurology.

Surgery, emergency, and psychiatry groups were not aggregated.

A binary measure of lifetime exposure to guns was created to categorize respondents as having ever had any of a variety of types of exposure to guns. Respondents selected all that apply to the question “Over the course of your life, what has been your involvement with firearms?” Response options were “There were guns in the house where I grew up,” “I used a firearm as part of my military service,” “I have fired a gun in the last year,” “ I own at least one handgun, rifle or shotgun,” “Someone else keeps guns in the house on or on the property where I live,” or “None of the above.” Respondents were also asked whether or not they had ever taken a course in the safe handling and use of firearms (other than as part of military service).

Respondents who selected any affirmative response to the lifetime exposure question (“Over the course of your life, what has been your involvement with firearms?”) and/or reported to have taken a course in safe handling and use of firearms were classified as having had lifetime exposure to firearms.

Respondents were asked to answer the question “When you do NOT ask patients about access to guns, to what degree do the following reasons keep you from asking?” on a scale from 0 to 100. We coded responses of 0 as “Not at all,” 1-25 as “A little,” 26-50 as “A moderate amount,” 51-75 as “A lot,” and 76-100 as “A great deal.” We transformed these responses into a binary variable to compare those who reported the degree to which a reason kept them from asking as “not at all” or “a little” with those who reported the item as at least a moderate reason for not asking (“a moderate amount” + “a lot” + “a great deal”)--that is, they found the reason a “notable barrier” to asking.
